# Phospho-iTRAQ data article: Assessing isobaric labels for the large-scale study of phosphopeptide stoichiometry

**DOI:** 10.1016/j.dib.2015.04.012

**Published:** 2015-05-06

**Authors:** Pieter Glibert, Paulien Meert, Katleen Van Steendam, Lennart Martens, Dieter Deforce, Maarten Dhaenens

**Affiliations:** a Laboratory of Pharmaceutical Biotechnology, Ghent University, B-9000 Ghent, Belgium; b Department of Medical Protein Research, VIB, B-9000 Ghent, Belgium; c Department of Biochemistry, Ghent University, B-9000 Ghent, Belgium

## Abstract

The ability to distinguish between phosphopeptides of high and low stoichiometry is essential to discover the true extent of protein phosphorylation. We here extend the strategy whereby a peptide sample is briefly split in two identical parts and differentially labeled preceding the phosphatase treatment of one part (Pflieger et al., 2008. *Mol. Cell. Proteomics*, 7: 326–46 [1]; Wu et al., 2011. *Nat. Methods*, 8: 677–83 [2]). Our Phospho-iTRAQ method focuses on the unmodified counterparts of phosphorylated peptides, which thus circumvents the ionization, fragmentation, and phospho-enrichment difficulties that hamper quantitation of stoichiometry in most common phosphoproteomics methods. Since iTRAQ enables multiplexing, simultaneous (phospho)proteome comparison between internal replicates and multiple samples is possible. The technique was validated on multiple instrument platforms by adding internal standards of high stoichiometry to a complex lysate of control and EGF-stimulated HeLa cells. To demonstrate the flexibility of PhosphoiTRAQ with regards to the experimental setup and data mining, the proteome coverage was extended through gel fractionation, while an internal replicate measurement creates more stringent data analysis opportunities. The latter allows other researchers to set their own threshold for selecting potential phosphorylation events in the dataset presented here, depending on the biological question or corroboration under investigation. The latest developments in MS instrumentation promise to further increase the resolution of the stoichiometric measurement of Phospho-iTRAQ in the future. The data accompanying the manuscript on this approach (Glibert et al., 2015, *J. Proteome Res.***14**: 2015, 839–49 [5]) have been deposited to the ProteomeXchange with identifier PXD001574.

Specifications Table

**Table:** 

Subject area	*Biology, Chemistry*
More specific subject area	*Phosphoproteomics and mass spectrometry* (*MS*)
Type of data	*MS data and annotations*
How data was acquired	*MS: Data-dependent acquisition acquired on three different MS instruments: SynaptG2Si* (*Waters*)*, TripeTOF 5600* (*Sciex*) *and QExactive* (*Thermo*)
Data format	*Raw* (*.raw, *.wiff and *.raw, respectively)*, mgf peak lists,* *.dat and *.XML identified files from Mascot (*Matrix Science*)
Experimental factors	*HepG2 cells were treated or not with EGF and after tryptic digest spiked with an internal standard of isotopically heavy AQUA phosphopeptides.*
Experimental features	*The respective proteomes were fractionated using 1DPAGE, followed by tryptic digest. Peptides were briefly split and each half labeled with a different iTRAQ label before enzymatic dephosphorylation of one half and subsequent LC–MS analysis of the merged peptide mixture.*
Data source location	*Gent, Belgium*
Data accessibility	*Deposited to the ProteomeXchange with identifier PXD001574* (*http://proteomecentral.proteomexchange.org/cgi/GetDataset?ID=PXD001574*)

Value of the data

• As a new approach to quantify the stoichiometry of phosphorylation events, “Phospho-iTRAQ” is applicable to a wide range of biological models.
• This first “Phospho-iTRAQ” dataset is generated on MS instruments from three different vendors, illustrating that the approach can be applied platform-independent.
• As described in detail in the accompanying manuscript (*DOI: 10.1021/pr500889v*), a new data analysis approach is presented that uses internal duplicate measurements unique to this experimental workflow. It allows mining data at different thresholds. Depending on the biological question under investigation, other investigators can choose to mine the presented dataset at the threshold of their picking.

## 1 Experimental design, materials and methods

**Fig. 1:**
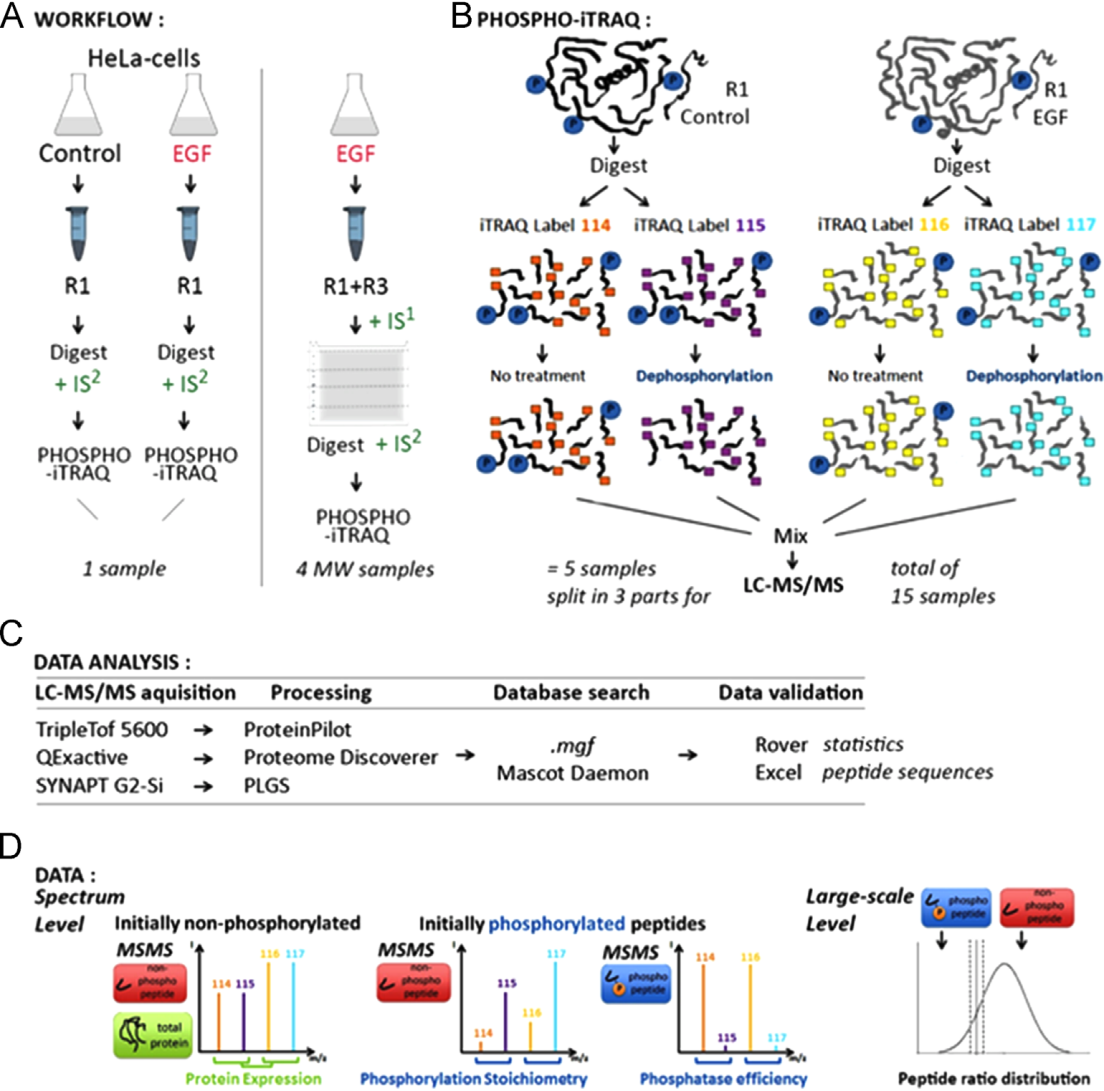
Experimental design of the Phospho-iTRAQ approach.

Fig. 1 shows the general workflow of the Phospho-iTRAQ approach. (A) Experimental workflow: in the “in-solution” approach (left panel), the soluble protein extracts (R1) of control and EGF-stimulated HeLa cells were spiked with an internal peptide standard (IS2) of heavy phosphopeptides and compared by Phospho-iTRAQ. When only one sample is mined (EGF), the flexibility of the protocol allows for gel purification and fractionation and thus for complementing the soluble protein extract (R1) with the hydrophobic fraction diluted in strongly denaturing buffer (R3) to increase the number of annotated phosphopeptides (“in-gel” approach, right panel). The EGF-stimulated cells were spiked with a phosphoprotein internal standard (IS1) before fractionation on a 1D PAGE into four molecular weight fractions followed by Phospho-iTRAQ. (B) PhosphoiTRAQ protocol: a peptide sample is briefly split in two identical parts and differentially labeled preceding the phosphatase treatment of one part. Afterward, samples are immediately recombined and split into three parts for the LC–MS/MS analysis on three different instruments. (C) Data Analysis: raw data was processed by the respective vendor׳s software, and MGF-files were searched against the SwissProt Human database using Mascot. Exported DAT-files were imported into Rover for ranking of the iTRAQ ratios and were further analyzed in Excel. (D) Data: initially phosphorylated peptides have skewed iTRAQ ratios and arise out of the center of the log-normal distribution of the whole precursor population. The mean of the log-normal ratio distribution is located around zero since the vast majority of the peptides in the data have equal 114/115 or 116/117 reporters.

### 1.1 Cell culture and lysis

HeLa cells were cultured at 37 °C in Dulbecco׳s Modified Eagle Medium (5% CO2) supplemented with 1% (w/v) l-glutamine, 10% (w/v) FBS and 50 IU/ml penicillin/streptomycin. Cells were starved in FBS-free medium 17 h before harvesting. For the EGF stimulation, the medium was removed, enriched with 150 ng/ml EGF and immediately re-added to the cells for 10 min. Next, the EGF-stimulated and control cells were incubated 15 min at 37 °C with PBS-based dissociation buffer (Invitrogen) and detached by cell scraping. Cells were washed twice by pre-cooled PBS and centrifugation at 4 °C. All subsequent steps were also performed at 4 °C unless declared otherwise. Cell lysis was performed by suspending the cell pellet in the R1-buffer from the Readyprep sequential extraction kit at a density of 1 ml/106 cells. R1 is comprised of 40 mM Tris-buffer which we supplemented with: 2% (v/v) Phosphatase Inhibitor Cocktail 2&3 (P5726 and P0044, Sigma-Aldrich), 2% (v/v) TBP, 0.3% (v/v) benzonase (E1014, Sigma-Aldrich) and 1 tablet/10 ml R1 cOmplete Mini EDTA-free Protease Inhibitor Cocktail (118361700001, Roche, Penzberg, Germany). After sonication in an ice-bath for 10 min (Branson 2510 sonication bath), the protein extracts were mixed with 25:1 volumes pre-cooled acetone for overnight incubation at −20 °C. The next day, after a centrifugation step at maximum speed (14.000 rpm) for 15 min, the acetone was removed and the pellet was resuspended in 0.5 M TEABC. After an additional spin, the pellet was removed and the supernatant was withheld as the “R1 extract”. A two-step lysis method was performed to the EGF-treated cells before gel fractionation, thereby extending the “Phospho-iTRAQ” method to increase the protein coverage. The protein extract of the cells in the R1-buffer was centrifuged at a maximum speed. To include more proteins the pellet was resuspended in the R3-buffer (containing urea, thiourea, detergents and ampholytes) from the Readyprep sequential extraction kit and combined with the initial R1 lysate, together forming the “R1+R3 extract”. The protein content of all the extracts was determined by the 2-D Quant Kit (GE Healthcare, Little Chalfont, United Kingdom).

### 1.2 Gel fractionation and digest

20 µg of the “R1 extract” from both the EGF-stimulated and control cells were digested analogous to the iTRAQ protocol (ABSciex). 500 µg of the “R1+R3 extract” derived from the EGF-stimulated cells was diluted 3:1 in 4x Laemmli Sample Buffer and reduced with 10% (v/v) BME for 10 min at 95 °C. Next, 1.5% (w/w) of the PeppermintStick internal protein standard (IS1) was added and the sample was loaded in the central well of the 10% Criterion Tris–HCl Gel, 11 cm IPG+ 1well. Electrophoresis of the gel was performed for 30 min at 150 V and 60 min at 200 V in a Criterion Cell (Biorad) and monitored by the Precision Plus Protein Marker in the outer well. After a fixation and washing step, the gel was cut into four equal pieces comprising different molecular weight fractions which were transferred to glass tubes. The proteins were in-gel digested according to optimized conditions [3]. All the obtained peptides were brought to dryness by the SpeedVac and stored at −20 °C until further use.

### 1.3 Labeling

Peptides of the “R1 extracts” were re-dissolved in 40 µl 50 mM TEABC, while peptides from the molecular weight fractions of the “R1+R3 extract” were re-dissolved in 80 µl. As a second internal standard (IS2), ±7 pmol of each phosphopeptide from the Heavy MS PhosphoMix 1, 2 and 3 (MSP1H, MSP2H, MSP3H Sigma-Aldrich) was spiked into each sample. Subsequently, “R1 extracts” were equally divided in two parts (Fig. 1), and the fraction of the “R1+R3 extract” was divided in four parts of 20 µl. Before the iTRAQ labeling, each reporter was reconstituted in 250 µl ethanol and combined with the related label of a second 4-plex set. For the ”R1 extracts”, the two halves of the control sample were labeled with 50 µl of the 114 and 115 reporter, the two halves of the EGF-stimulated sample with the 116 and 117 reporter, and each quarter of the four molecular weight fractions of the “R1+R3 extract” with 50 µl of a different 4-plex label. The labeling was performed for two hours at room temperature by continuous shaking before samples were dried out.

### 1.4 Dephosphorylation

The labeled peptides in the 20 vials (both R1 extracts were split in two, and each gel fraction of the R1+R3 extract was split in four) were re-dissolved in 12 µl calf intestinal phosphatase (CIP) buffer (100 mM NaCl, 50 mM Tris–HCl, 10 mM MgCl2, 1 mM DTT) and incubated for 45 min at room temperature in order to remove the residual free labels. Next, the 114–116 and 115–117 labeled peptides were combined, resulting in 10 vials (114–116 and 115–117 for the ”R1 extracts” as well as the four molecular weight fractions of the “R1+R3 extract”). To the 115–117 samples, 30 µl of the dephosphorylating enzyme cocktail (6,25% 0.25 U/µl Escherichia coli alkaline phosphatase (P4069, Sigma-Aldrich), 31.25% 10 U/µl calf intestinal phosphatase 1 (M0290, New England Biolabs), 31.25% 10 U/µl calf intestinal phosphatase 2 (P4978, Sigma-Aldrich)) and 31.25% 1 U/µl thermosensitive alkaline phosphatase (M9910, Promega)) was added. For the 114–116 control samples, 30 µl of CIP buffer was supplemented before all samples were incubated at 37 °C for the overnight dephosphorylation. Phosphatases were inactivated the next day by heating the samples for 30 min at 85 °C in the presence of EDTA (50 mM). The 114–116 and 115–117 units were merged and the five samples were cleaned by C18 tips (Sigma-Aldrich) to desalt and to remove free labels and excess of phosphatases. The peptide samples which were eluted with 80% acetonitrile (ACN), 20% water, 0.1% formic acid (FA), were spilt into three equal parts and were dried out immediately.

### 1.5 LC–MS/MS analysis

Peptides were resolved in 0.1% FA and approximately 0.5–1 µg was brought on column each run. The nanoLC-MS/MS analysis of the three parts was performed on different ESI mass spectrometry platforms: the first part on a TripleTof 5600 (ABsciex), the second part on a QExactive (Thermo Fisher) and a third part on a SYNAPT G2-Si (Water Cooperation). 1) For the analysis on the TripleTof 5600, the chromatography was done on an Eksigent ekspert^™^ nanoLC 400 System on NanoLC column, 3 μm, ChromXP C18CL, with a 120 min gradient going from 5% to 90% ACN, 0.1% FA and a 300 nL/min flow. During acquisition, each survey scan accumulated precursors in the range of 400–1250 m/z for 250 ms from which the top 20 were fragmented for MS/MS every cycle at a ratio of 180 ms/precursor. Resolution was>15k in MSMS; dynamic exclusion 10 s. 2) The QExactive was coupled to a Dionex Ultimate^™^ 3000 RS nanoLC System. After trapping on a pre-column, peptides were separated on the Acclaim PepMap100, 75 μm×50 cm by a 90 min gradient whereby the amount of ACN 0.1% FA increased from 5 to 70%, also at a constant flow rate of 300 nl/min. In a scan range of 380–2000 m/z, a Full MS and data dependent MS/MS scan was performed on the top 10 by a normalized collision energy (NCE) 30 stepped+collision energy 10% in all cases. Resolution was>25k in MSMS; dynamic exclusion 25 s. 3) For the measurements on the SYNAPT G2-Si, a NanoACQUITY^™^ system separated the samples with a Waters BEH C18, 75 μm×150 mm analytical column after a trapping step of 8 min through changing the amount of ACN, 0.1% FA changed from 1 to 40% for 90 min at a constant flow rate of 300 nl/min. The range of 400–1600 m/z was inspected during the 0.1 s survey scan from which the top ten of the precursors were fragmented by ramping the collision energy in the “iTRAQ” mode for a 0.1 s/MS/MS scan if the threshold of 10k counts/spectrum was reached. Resolution was>20k in MSMS; dynamic exclusion 30 s.

### 1.6 Data analysis

Data acquisition, recording and pre-processing was carried out with software of the respective mass spectrometer manufacturers. Equally, the raw data was processed by specialized software packages of the vendors: ProteinPilot^™^ 4.0.8085 for the TripleTof 5600 of ABSciex, Proteome Discoverer 1.4 for the Thermo Scientific QExactive, and PLGS 3.0.1 for the SYNAPT G2-Si of Waters Cooperation. The same software packages were used to export the created peak lists in the MGF format. The database searches of these MGF files were performed with Mascot 2.4.0. (Matrix Science) against the SwissProt Human database (59.084 sequences, supplemented with the sequences of the internal standards). Initially, the prevalence of the most common modifications was explored by an Error Tolerant database search (25). Details an search parameters can be found in the ⁎.dat files. The result files of these searches were exported as Mascot-DAT file formats (Peptide e-value<0.05), and each run was then imported separately in the Rover software for individual processing (https://code.google.com/p/compomics-rover/) (26). After obtaining Rover statistics on the 114/115 and 116/117 ratios, the data were filtered in order to export the log 2 ratios and z-scores of unique or “razor” peptides for consecutive data validation. Data validation and frequency plot distribution of Z-scores (at a bin size of 0.1) was done in Excel, whereby the Z-scores of the four molecular weight fractions of the “R1+R3 extracts” were combined. Additional database searches were performed by changing the variable modifications in order to annotate the “heavy” internal standard phosphopeptides.

Phosphoprotein enrichment in the obtained protein lists was assessed by a right-sided Fisher enrichment test at a significance level of 5%. Known phosphoproteins in human were obtained from the PhosphoSitePlus database (http://www.phosphosite.com). The total number of proteins known in human was estimated based on the described proteins in the PFAM database. Proteins involved in the EGF pathway were identified by querying the BioCarta database.

To assess the relative amount of phosphopeptides present in the lists generated by the Phospho-iTRAQ approach, these lists were compared to the known phosphopeptides present in the PhosphoSitePlus database by means of a protein blast as integrated in the NCBI blast 2.2.28 package. The procedure allowed for partial mappings between Phospho-iTRAQ peptides and the phosphopeptides. That is, stretches of at least 5 amino acids in both peptide sequences had to map unambiguously to each other (irrespective of overhangs that result from e.g. tryptic miscleavages), i.e. no gaps or mutations were allowed in the procedure.

The mass spectrometry proteomics data have been deposited to the ProteomeXchange Consortium [4] via the PRIDE partner repository with the dataset identifier PXD001574 and 10.6019/PXD001574.
